# Assessing the sensitivity and representativeness of the Belgian Sentinel Network of Laboratories using test reimbursement data

**DOI:** 10.1186/s13690-016-0145-9

**Published:** 2016-08-08

**Authors:** Nicolas Berger, Gaetan Muyldermans, Yves Dupont, Sophie Quoilin

**Affiliations:** 1 WIV-ISP (Scientific Institute of Public Health), Avenue de la Couronne, 310, 1050 Brussels, Belgium; 2 Department of Social and Environmental Health Research, London School of Hygiene and Tropical Medicine, 15-17 Tavistock Place, London, WC1H 9SH UK

**Keywords:** Epidemiology, Surveillance system, Sentinel network, Coverage, Representativeness

## Abstract

**Background:**

The Belgian Sentinel Network of Laboratories (SNL) was created in 1983 in order to monitor trends in infectious diseases. Given the evolution of the surveillance system, such as the waivers, fusions and adhesions of laboratories over time, it is important to evaluate whether the SNL is still fit for purpose. This study aims to evaluate aspects of the sensitivity and representativeness of the SNL by means of a test coverage analysis.

**Methods:**

We estimated test coverage of the SNL using the ratio of reimbursed tests performed by participating laboratories to the total number of tests performed between 2007 and 2012, for 12 (groups of) pathogens. We further evaluated the geographical difference coverage of the SNL at regional and provincial levels.

**Results:**

We found that test coverage of the SNL was stable over time and close to, or greater than, 50 % for the 12 (groups of) pathogens studied. These results hold for the three regions of Belgium but not for all provinces. We showed that some provinces had a low test coverage for some pathogens and that test coverage was more variable over time at provincial level.

**Conclusions:**

This sensitivity and representativeness study based on test coverage suggests that the SNL is capable to describe trend and to monitor changes in the 12 (groups of) pathogens studied both at national and regional levels. Therefore, the SNL is useful to contribute to estimate the burden of disease and to inform preventive measures. It should however be reinforced to allow to be used as an alert system at provincial level.

## Background

In spite of the rising contribution of noncommunicable diseases to the global burden of disease, communicable diseases continue to threaten population health in high income countries. Public health authorities seek to evaluate this threat. In Belgium, a surveillance system for communicable diseases has been established in 1983 on the basis of a voluntary Sentinel Network of Microbiological Laboratories (SNL) [1]. The Scientific Institute of Public Health (WIV-ISP) coordinates the SNL and weekly gathers laboratory-diagnosed cases reported by participating laboratories for more than 30 infectious diseases. The surveillance system aims to monitor trends in communicable diseases in order to be able to inform and alert health authorities on the current epidemiological situation. Its objectives include the uncovering of geographical and seasonal variations, the detecting of emerging threats [2], as well as the appraisal of the effectiveness of preventive measures such as vaccination [3].

The SNL has evolved over time. Since its creation, the surveillance system has adapted to the structural reorganisations, fusions, waivers and adhesions of laboratories; it has accounted for the use of new diagnosis methods such as PCR amplification; it has integrated European case-definitions; and it has encouraged laboratories to modernise their reporting method, moving from paper-based reporting to Electronic Data Interchange. Whereas some of the changes have had a positive impact on the quality of the surveillance, others might have altered the ability of the SNL to fulfil its missions. It is therefore important to regularly evaluate whether the surveillance system is fit for purpose [4].

To be able to detect changes in the epidemiological situation, the surveillance system should cover a suitably large and representative proportion of the Belgian population, and ensure that the population under study remains constant over time [5, 6]. These characteristics are embraced by two closely related attributes of surveillance systems: sensitivity and representativeness. In the context of surveillance systems evaluation, sensitivity, in addition to being a synonym of true positive rate, refers to the ability of a surveillance system to monitor changes in the number of cases over time and to detect outbreaks [6]. Representativeness indicates whether a surveillance system accurately describes the occurrence of events under surveillance over time, person and place [6]. Of particular importance is the ability to equally capture all infectious diseases under surveillance and to reflect non-homogeneous distributions of diseases across place. Some aspects of sensitivity and representativeness can be evaluated using coverage measures which indicate the proportion of the target population included in the surveillance system. Ideally, the coverage of a surveillance system should be constant over place and across pathogens to allow to detect regional outbreaks or specificities, and stable over time to allow meaningful interpretation of the time series.

The coverage of the SNL was annually monitored using the ratio of participating laboratories to the total number of laboratories. Data indicate constant ratios over the last 10 years, close to 60 %. The ratios were constant across Flanders, Wallonia and Brussels, which gives some indication of the geographical representativeness of the SNL. However, these figures are poor proxies of the coverage of the SNL because the laboratories have different catchment populations and greatly vary in terms of size and activity. A better measure of coverage was presented by Vandenberghe who analysed laboratory test reimbursement data to estimate test coverage of 15 pathogens. The study revealed that participating laboratories were performing more than 50 % of pathogen-specific tests, and that the test coverage was constant between 1999 and 2002 [7]. The study also indicated that participating laboratories were more often connected to a hospital and tended to conduct more tests as compared to non-participating laboratories, but were similar with respect to other characteristics. It is unknown whether these differences could lead to systematic bias.

Given the evolution of the SNL since 2002, little is known on whether the coverage of the network remained constant over the last years. In addition no studies have evaluated whether the SNL was representative across place, beyond the three regions of Belgium.

This study aims to evaluate aspects of the sensitivity and representativeness of the SNL by analysing test coverage, its geographical distribution, its consistency across pathogens and its stability over time. We estimated the coverage of the SNL using microbiology tests reimbursement data between 2007 and 2012, for 12 pathogens or groups of pathogens.

## Method

### Data

Data on reimbursed microbiology tests were obtained from the Belgian National Institute for Health and Disability Insurance (INAMI-RIZIV) for the period 2007–2012. The INAMI-RIZIV database contains all microbiology tests for which laboratories have claimed reimbursement to the compulsory national social security system. The database is therefore virtually exhaustive, both over time and place, with the unlikely exception of tests being performed without being reimbursed. Microbiology tests are identified with a nomenclature number that serves for reimbursement purposes. We also tracked changes in the INAMI-RIZIV nomenclature over time. We identified infectious diseases monitored by the SNL for which a specific reimbursement test was available in the INAMI-RIVIZ nomenclature [8]. Specific reimbursement codes (ambulatory and hospital numbers) were found for 8 pathogens - *Borrelia*, *Chlamydia trachomatis*, *Cryptosporidium*, *Neisseria gonorrhoeae*, *Hepatitis A*, *Mycoplasma pneumonia*e, *Rotavirus*, and *Syphilis* (*Treponema pallidum*) - and a group of enteric infections including *Salmonella*, *Shigella*, *Yersinia* and *Campylobacter* (Table 1). Due to privacy concerns, the INAMI-RIZIV did not provide the number of tests performed for each laboratory, but rather aggregated the number of tests by arrondissement. To obtain the data, we provided the INAMI-RIZIV with a list of laboratories, their INAMI-RIZIV certification code, whether or not they belonged to the SNL and their postcode. A list was created for each year in order to account for changes in the accreditation codes (e.g. following the fusion of laboratories) and in the participation status to the SNL. We defined annual participation of a laboratory if it had reported at least one pathogen to the SNL for a given year. For each year between 2007 and 2012, we obtained the number of reimbursed test by nomenclature code and arrondissement, separately for laboratories participating and not participating to the SNL. For any nomenclature code, less than 1 % of the tests failed to be paired with a laboratory accreditation code. These tests were excluded assuming missingness completely at random.

**Table 1:** Diagnosis tests by pathogen in the Belgian National Institute for Health and Disability Insurance nomenclature (INAMI-RIZIV)

Pathogen	Diagnosis test	Nomenclature number	
		Ambulatory	Hospital
*Borrelia*	Search for anti-Borrelia IgG antibodies	551132	551143
	Search for anti-Borrelia IgM antibodies^a^	552134	552145
	Search for anti-Borrelia IgG antibodies, in CSF^a^	552156	552160
	Search for anti-Borrelia IgM antibodies, in CSF^a^	552171	552182
	Search for anti-Borrelia IgG antibodies, immunoblot confirmation test^a^	552193	552204
	Search for anti-Borrelia IgM antibodies, immunoblot confirmation test^a^	552215	552226
	Search for anti-Borrelia IgG antibodies in CSF, immunoblot confirmation test^a^	552230	552241
	Search anti-Borrelia IgM antibodies in CSF, immunoblot confirmation test^a^	552252	552263
*Chlamydia trachomatis*	Chlamydia culture	550675	550686
	Chlamydia molecular amplification	550255	550266
*Cryptosporidium*	Search for cryptosporidium in stool, after enrichment	549872	549883
*Neisseria gonorrhoeae*	Gonorrhoeae molecular amplification	550911	550922
	Aerobic culture	550395	550406
*Hepatitis A*	Search for anti-Hepatitis A IgM antibodies, non-isotopic method	551353	551364
	Search for anti-Hepatitis A IgG antibodies	551375	551386
*Mycoplasma pneumoniae*	Search for anti-Mycoplasma pneumoniae IgM antibodies	551891	551902
	Search for anti-Mycoplasma pneumoniae IgG antibodies	551213	551224
*Rotavirus*	Rotavirus diagnosis, for children younger than 2 years old	552311	552322
*Salmonella/Shigella/Yersinia/Campylobacter*	Stool test	549835	549846
*Syphilis (treponema pallidum)*	Serodiagnosis of treponema	552716	552720

^a^ No reimbursement number before April 2008

### Analysis

Reimbursement data are used to assess aspects of the sensitivity and representativeness of the SNL by looking at different measures of test coverage. In the absence of information on the true number of (diagnosed) cases in the Belgian population, we used reimbursement data as a way of proxying case coverage [7]. Test coverage is the ratio between the number of tests reimbursed by laboratories of the surveillance system and the total number of tests reimbursed. A high test coverage value indicates that most of the tests performed are captured by the surveillance system, and therefore, that cases are more likely to be reported to the surveillance system. Conversely, a low test coverage value indicates that most of the tests are performed by laboratories that do not take part to the surveillance system and therefore, that cases are less likely to be reported. We examine the test coverage of the SNL by pathogen at national level, its variations by region and province and the stability of the coverage over time for the period 2007–2012. These levels of analysis allow to inform on representativeness by time and place and to indicate whether pathogens are equally monitored. The extent and stability of test coverage values by place and time also informs on sensitivity, i.e. the ability to monitor change over time at different geographical levels. It is important to note that this study is not based on a sample but includes the total number of tests reimbursed in Belgium for the selected pathogens. Therefore, we report descriptive statistics and do not attempt to make statistical inference. Coverage values are graphically represented to allow to visually identify variations over pathogen, place or time.

## Results

### Number of tests reimbursed by pathogen

**Fig. 1:**
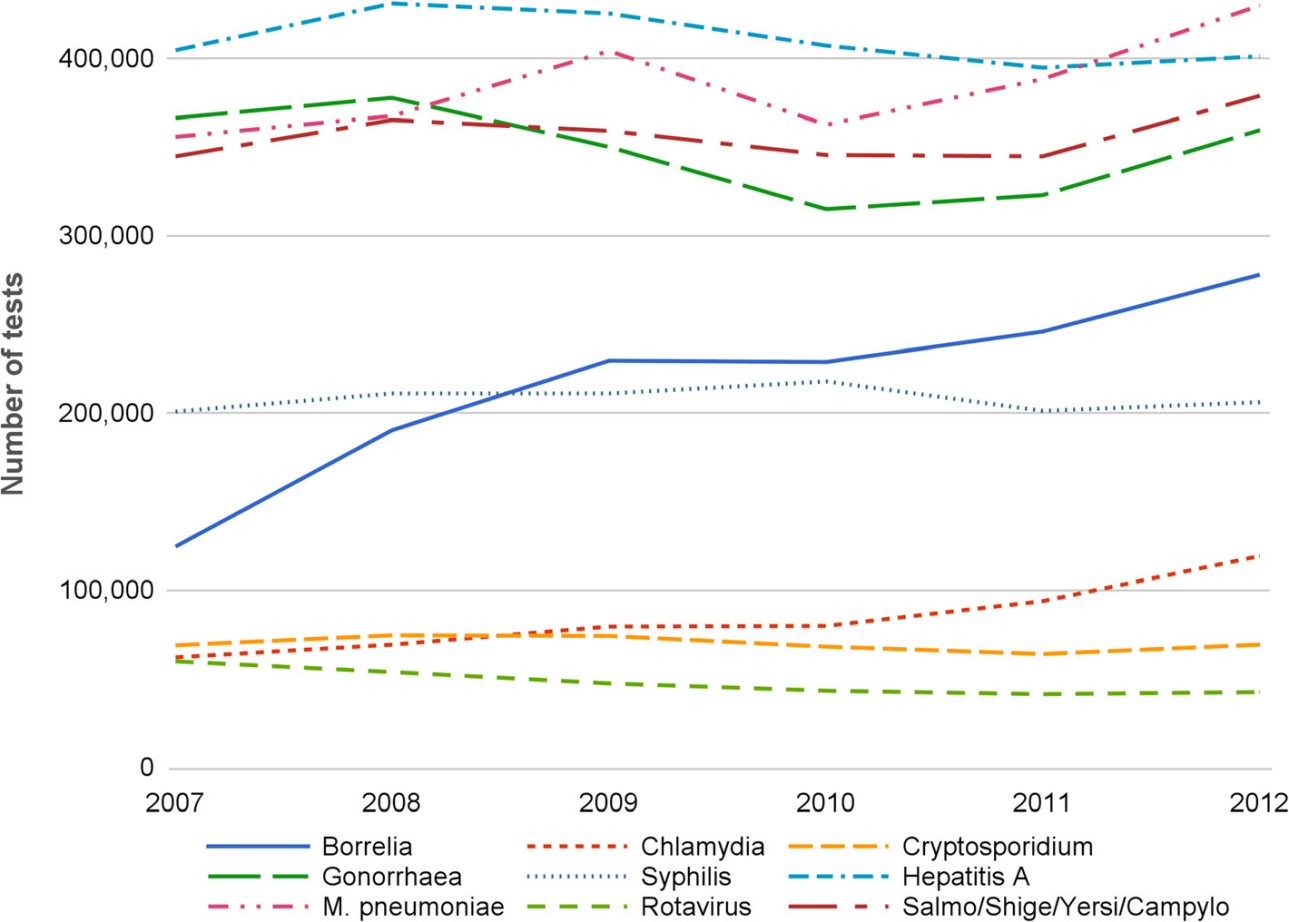
Evolution of the number of reimbursed tests by (group of) pathogen in Belgium (2007–2012)

The number of tests reimbursed by the INAMI-RIZIV increased during the period 2007–2012 (Fig. 1). In 2007, slightly less than 2 million tests were performed to detect one of the 12 pathogens under study. The number of tests increased until 2009, then slightly decreased in 2010, and increased again to reach a value of almost 2.3 million in 2012. The increase is partly due to a growth of reimbursement for *Borrelia* tests during this period. The introduction of a new nomenclature code for *Borrelia* in 2008 (Table 1) has led the number of reimbursed tests to rise from 124,403 in 2007 to 277,805 in 2012. Important increase in the number of reimbursed tests is also observed for *M. pneumoniae* and *Chlamydia*; whereas the figures for the group of enteric infections (*Salmonella*, *Shigella*, *Yersinia*, *Campylobacter*), *Rotavirus* and *Hepatitis A* fluctuate over time.

Data also reveal pathogen variations in the number of tests. Fewer tests are reimbursed for *Rotavirus*, *Chlamydia* and *Cryptosporidium* (less than 100,000 annually) as compared to *M. pneumoniae*, *Hepatitis A*, *Gonorrheae* and enteric infections (more than 300,000 annually).

### National test coverage

**Fig. 2:**
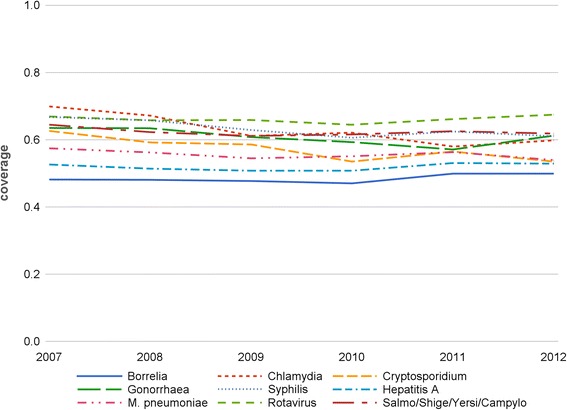
Evolution of the test coverage of the Sentinel Laboratory Network by (group of) pathogen in Belgium (2007–2012)

In 2012, the SNL performed the majority of the reimbursed tests (Fig. 2). Test coverage was high for all (groups of) pathogens and ranged from 49.9 % (*Borrelia*) to 67.5 % (*Rotavirus*). Coverage was stable and ranged between approximately 50 and 70 % in the 2007–2012 period. Average coverage was highest in 2007, decreased a little until 2010 (see Cryptosporidium), and increased afterwards to reach a mean value of 58.0 % in 2012 (median 59.9 %).

### Regional test coverage

**Fig. 3:**
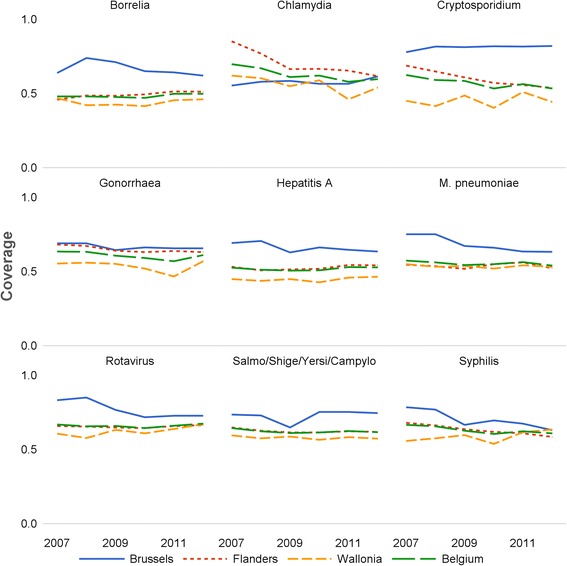
Evolution of the test coverage of the Sentinel Laboratory Network by (group of) pathogen and region of Belgium (2007–2012)

Coverage was high in the three regions and relatively stable over the whole period (Fig. 3). Between 2007 and 2012 the test coverage of the SNL was highest in Brussels and lowest in Wallonia for most pathogens. In Flanders, coverage was close to the national level. In 2012, it ranged from 61.5 to 82.2 % (median 63.7 %) in Brussels, from 51.2 to 66.6 % in Flanders (median 58.7 %), and from 44.3 to 67.1 % in Wallonia (median 54.1 %). Coverage varied slightly more over time in Brussels than in the other regions. In Flanders and Wallonia, coverage was constant over time for most (groups of) pathogens, with some yearly variations for *Chlamydia* and *Cryptosporidium*. Overall, results indicate high and constant test coverage values by (group of) pathogen, region and year.

### Provincial test coverage

**Fig. 4:**
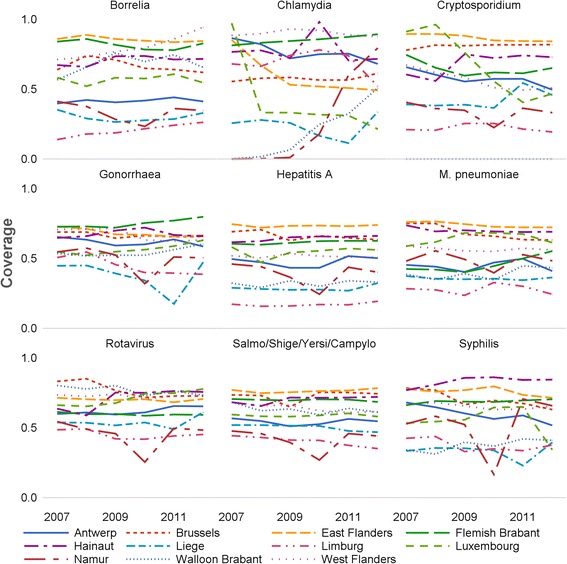
Evolution of the test coverage of the Sentinel Laboratory Network by (group of) pathogen and province of Belgium (2007–2012)

Variation in coverage was larger at provincial level (Fig. 4). Coverage was globally lower in Namur, Walloon Brabant, Liege and Limburg over the 2007–2012 period. Some pathogens (*Borrelia*, *Chlamydia* and *Cryptosporidium*) indicate great coverage variability between provinces. For instance, the coverage of *Cryptosporidium* lied above 90 % in East Flanders and is null in Walloon Brabant. Other pathogens, showed varying coverage levels over time for specific provinces, as it is the case for *Chlamydia* for which we observe a dramatic increase in coverage in Namur and Walloon Brabant after 2009. Overall, coverage varied by (group of) pathogen at provincial level, but was rather constant over time, with a few exceptions.

## Discussion

We found that test coverage of the SNL was stable over time and close to, or greater than, 50 % for the 12 pathogens or groups of pathogens studied. These results hold for the three regions of Belgium. For the first time, this study also allowed to investigate provincial variations in test coverage. We showed that test coverage was constant over time in most provinces, despites a greater variability in the values.

Results indicate that the SNL is sensitive and representative for the surveillance of the selected infectious diseases. Effectiveness of vaccination strategies, seasonal variations, outbreaks and all sorts of variations in the occurrence of the pathogens are very likely to be captured by the surveillance system if they occur at a national or regional level. The SNL is nevertheless less likely to capture local changes over time in some provinces such as Namur, Walloon Brabant, Liege and Limburg.

Variability over time observed in some provinces could be accounted by changes in the participation status of laboratories and/or non-concordance between available accreditation numbers of laboratories and those used in the INAMI-RIZIV reimbursement database. The latter is likely to explain the sharp decrease in Namur in 2010 for example.

Regional figures are comparable to those described by the French Epibac Network in 2010, although the French network had a higher global coverage, close to 75 % [9]. Comparison with a former study [7] seems to indicate that the coverage slightly decreased for a few pathogens (e.g. for *Borrelia*, *Cryptosporidium* and *Hepatitis A*) since the period 1999–2002. Differences in the results might be explained by factors such as changes in the reimbursement nomenclature (e.g. *Borrelia*), changes in the testing behaviours and changes in the types of laboratories participating to the surveillance system (although the proportion of laboratories participating has increased, some laboratories did quit the network since 2002 and fusions have been observed). In addition, unlike Vandenberghe [7], we defined laboratories not sending data as inactive, even though they officially belonged to the SNL. Our coverage values are therefore expected to be slightly lower and closer to reality. Unfortunately, data do not allow to indicate whether the slight decrease is an artefact or is real and might therefore have an impact on the trends of notification rates monitored by the WIV-ISP.

This study has several limitations. First, it relies on the quality and exhaustively of the reimbursement data, without being able to evaluate it. It is indeed likely that some tests performed by laboratories to identified pathogens are not included in the INAMI-RIZIV database. Second, our approach identified laboratories using certification number for reimbursement. As laboratory identification codes evolve over time and between sources, some tests could not be identified in the list of laboratories codes provided to the INAMI-RIZIV to obtain reimbursement data. In addition, multiple laboratories associated or belonging to the same group may share a same laboratory code (e.g. after a fusion) while not all laboratories from the group participate to the surveillance network. In this study we assumed that if at least one laboratory participated to the SNL, all laboratories from the same group and with the same identification code were also participating. This assumption does not always hold which means that coverage values could be slightly biased in that respect. Third, data were only available for a short period of time and do not allow to assess how changes in test coverage of the SNL since the 1980s might influence the meaning and interpretation of the reported number of cases over time. Data were furthermore only available for 12 pathogens which are fairly well distributed over place and widely tested. It is unknown whether results hold for pathogens which are less often tested (such as *Hantavirus*) or which are more clustered or subject to outbreaks (such as *Hepatitis A*, *Legionella*, *Neisseria meningitis*, or *Listeria*). Testing behaviours are also likely to differ by pathogen. Some tests, such as *Syphilis*, are systematically prescribed for some subgroups (e.g. pregnant women). These will tend to be well reported to the SNL as they are mainly performed by laboratory hospitals, which are overrepresented in the SNL. In certain circumstances, a high test coverage, might therefore not translate into representativeness or sensitivity. Alternative surveillance systems are therefore available in order to adequately monitor infectious diseases which are not evenly distributed in the general population, such as sexually transmitted diseases.

In other instances, individuals might perform multiple tests to diagnose the same disease. This might lead to an overestimation of the representativeness of the SNL if these repeated tests are not randomly distributed between SNL and other laboratories. If, for example, an individual is diagnosed at multiple university hospitals, which are known to be more likely to participate to the SNL, the measure of coverage might slightly be overestimated. The SNL would however be able to identify duplicates amongst the reported cases.

This study proved to be useful in showing potential limitations of the SNL to monitor infectious diseases with uneven geographical distributions. Results indicate that better geographical representativeness and sensitivity could be obtained by recruiting new participating laboratories in the four provinces where lower test coverage was detected: Namur, Walloon Brabant, Liege and Limburg.

Another lesson learned from this study is that one should be cautious when interpreting data from the SNL. Analysing provincial notification rates or changes over time might for example not be relevant for some pathogens. Automatic representation of provincial data should therefore be avoided. Rigorous analysis should preferably rely on multiple sources of data, as recently illustrated by Sabbe et al. [3] who analysed the impact of vaccination on rotavirus activity, using a diversity of data sources such as SNL data, test reimbursement data and hospitalisation data.

In this study, we evaluated restricted aspects of the surveillance system and indicated that the SNL, by design, has sufficient precision and reflects the Belgian situation without important systematic bias. Many other characteristics of the SNL have an influence on the capacity of the surveillance system to effectively monitor infectious diseases. A wider evaluation of the surveillance system is therefore strongly recommended in order to have a better understanding of the actual quality of the data reported by the SNL [5]. For example, there might be systematic bias arising from the fact that some participating laboratories do not report all diagnosed cases, or only report cases for certain pathogens. Given the evolving nature of the SNL, the impact of changes in the network characteristics (type of data transfer, fusion of laboratories, evolution of pathogen detection techniques, cases definition) should be systematically documented and continuously assessed. Obtaining reimbursement data by laboratory, and not by province, is necessary for that purpose. National Reference Centre and Obligatory Notification data should also be used to evaluate the surveillance system.

Through this study, we illustrated the importance of evaluating (some) attributes of surveillance systems and showed that available reimbursement data could serve as a tool for improving the representativeness of a surveillance system in the absence of other data sources. Reimbursement data have been underused so far. In the future, one can think of using reimbursement data together with the number of reported cases in order to infer the incidence of a disease. Such a procedure would assume similar test positivity for participating and non-participating laboratories, an assumption which deserves to be further investigated. Having regular reimbursement data by laboratory would therefore both allow to monitor the quality of surveillance and open up new opportunities.

## Conclusions

This test coverage study suggests that the SNL is capable of describing the epidemiological situation and monitoring changes in the 12 pathogens or groups of pathogens studied both at national and regional levels. It should however be reinforced in four out of the 11 provinces to allow to be used as a sensitive alert system at provincial level.

## Abbreviations

INAMI-RIZIV, Belgian National Institute for Health and Disability Insurance; SNL, sentinel network of laboratory; WIV-ISP, Belgian Scientific Institute of Public Health.
